# Supplementary material for: On the Morphological Deviation in Additive Manufacturing of Porous Ti6Al4V Scaffold: A Design Consideration
Source: Materials (Basel). 2022 Jul 6;15(14):4729. doi: 10.3390/ma15144729 (PMC9319900; doi:10.3390/ma15144729)
Supplement: Supplementary file 1 [file materials-15-04729-s001.zip › materials-1786384-supplementary.pdf]

Measurements of strut thickness and pore size

Measurements of two variables, strut thickness (ST) and pore size (PS), were made for vertical struts and for horizontal struts in 2 types of 3D printed structures: diamonds and gyroids. There were 5 replicate samples of each structure, and within each replicate structure, each variable was measured at 30 different locations within each of 4 slices, which are vertical cross-sections approximately equally spaced across the structure. Different slices were used to measure vertical and horizontal struts, and different locations within slices were used to measure ST and PS. So there are $5 \times 4 \times 30 = 600$ measurements of ST and also of PS for each structure type and strut orientation.

Figure S1 shows the complete set of measurements for diamonds. The 30 individual measurements of ST and PS in each slice are plotted as blue dots against a common scale for each strut orientation, for each replicate structure separately. The mean values of the 30 measurements are marked as red triangles and their standard deviations are printed in red in each panel. The target values are marked by grey vertical lines. The strut thicknesses tend to be larger than the target value, and more so for horizontal struts. Correspondingly, the pore sizes tend to be smaller than the target, and more so for horizontal struts. Within each row of panels the distributions of measurements are broadly consistent and there is no apparent systematic dependence on slice order or replicate number. There are a number of relatively extreme high and low values (for example, more the 50 microns from their slice means) which could be relevant to the performance of the structure.

Figure S2 shows the complete set of measurements for gyroids in the same way. Again, the strut thicknesses tend to be larger than the target and the pore sizes tend to be smaller. The differences of the mean values from their targets are again larger for horizontal strut measurements, and the comparison between horizontal and vertical struts is even more pronounced than it is for diamonds. Furthermore, the scatter of the measurements about their slice means is much larger for horizontal struts resulting in more relatively extreme values. Again there is no apparent systematic variation with slice number or replicate number, except

possibly for Replicate 5 where the spread of the individual measurements is smaller than that for the other replicates.

Figure S3 shows histograms of the 600 individual measurements minus their slice means, pooled over all 20 slices for each group, along with fitted normal density functions. This figure reveals a peaked (leptokurtic) nature of the empirical distributions of the measurements with relatively longer tails compared with a Normal distribution. None of our analyses of the individual measurements require them to be normally distributed. However, the nature of their distribution may have implications for the performance of the structure.

Table S1 lists the means and standard deviations of the 30 measurements within each slice. The mean values are the basic data for the analysis of variation between slices. The standard deviations are plotted against the mean excess from target in Figure 6.

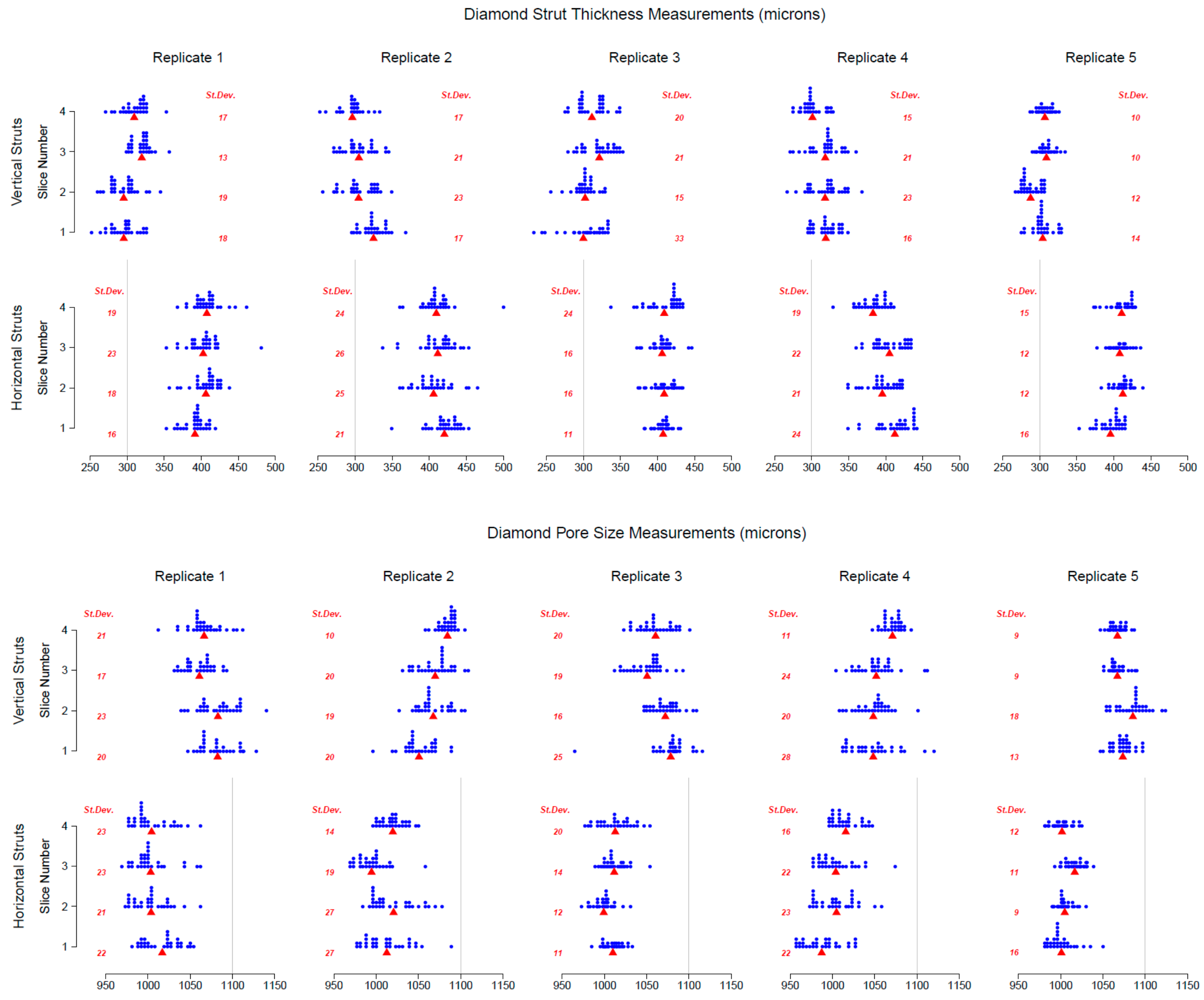

Figure S1. This shows the complete set of measurements for diamonds. The 30 individual measurements are plotted as blue dots against a common scale for each slice and strut orientation, for each replicate structure separately. The mean values of the 30 measurements are marked as red triangles and their standard deviations are printed in red in each panel. The target values are marked by grey vertical lines. The strut thicknesses in microns are shown in the upper two rows of panels, all with the same axis scale, and the pore sizes in microns are shown in the lower two rows, all with the same scale.

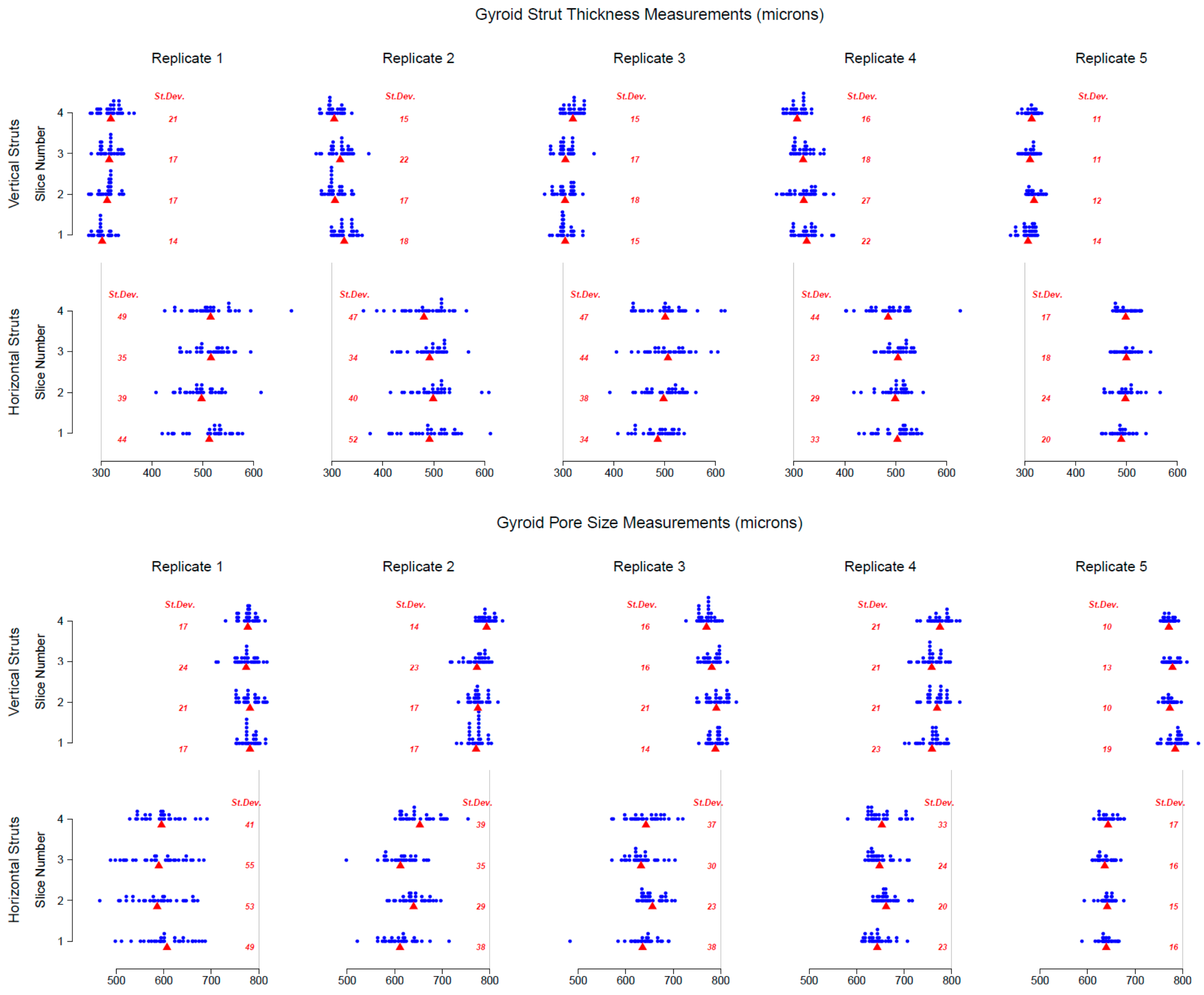

Figure S2. This shows the complete set of measurements for gyroids. The method of plotting and conventions are the same as in Figure S1.

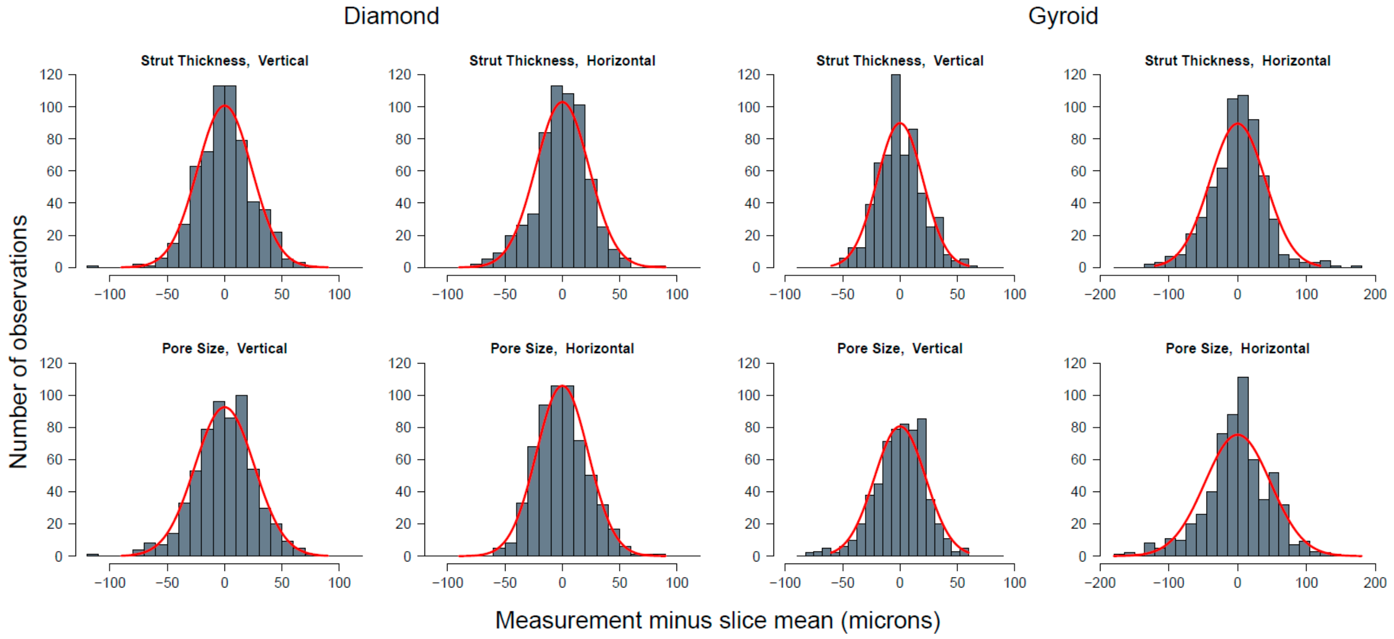

Figure S3. Histograms of the 600 individual measurements minus their slice means, pooled over all 20 slices, for each structure type, variable and strut orientation. The red curves are fitted Normal density functions with mean 0 and standard deviations s1 given in Table 3.

Distribution of the slice means and standard deviations

Table S1 lists the means and standard deviations of the 30 measurements within each slice. The standard deviations are plotted against the mean excess from target in Figure 6. The mean values are the basic data for the analysis of variation between slices. For the validity of the anova calculations it is not required that the slice means should be normally distributed about their respective population means. However, the *t*-test *p*-values and confidence intervals in Table 3 are formally based on that assumption, though they are robust to moderate departures from it. Figure S4 shows Normal Q-Q-plots of the 20 slice means for each group of measurements. For a sample from a Normal population, the points would tend to increase roughly in a straight line with intercept and slope given by the population mean and standard deviation. Figure S4 confirms that it is reasonable to assume that the slice means are normally distributed for the purpose of calculating *p*-values and confidence intervals from the t-

distribution. A formal test for normality would not be informative here, with a sample size of only 20.

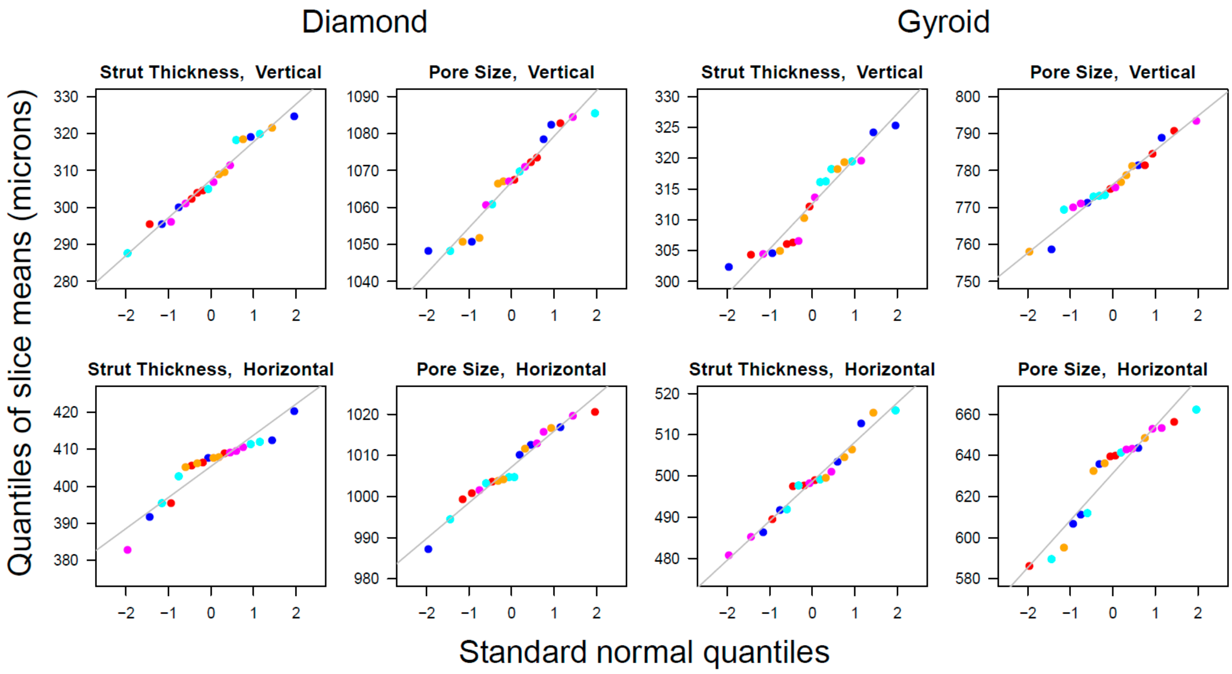

Figure S4. Normal Q-Q plots of the 20 slice means, for each structure type, variable and strut orientation. Different colours denote the 5 different replicates of each printed structure. The grey line in each panel has intercept and slope given by the mean and standard deviation of the 20 slice means.

Table S1. Means and standard deviations (in microns) of the 30 measurements within each slice within each replicate for each structure type, variable and strut orientation.

				Mean					Standard Deviation				
		Replicate Number		1	2	3	4	5	1	2	3	4	5
		Slice order											
Diamond	ST	Vertical	1	295.5	324.6	299.9	319.1	304.0	18.3	16.7	32.8	16.2	14.0
			2	295.4	304.5	302.2	318.1	287.6	19.3	23.0	15.3	23.0	12.2
			3	319.8	304.9	321.4	318.4	308.9	12.8	20.8	21.0	21.4	10.3
			4	309.6	296.0	311.4	301.1	306.8	16.8	16.6	20.2	14.9	9.7
		Horizontal	1	391.6	420.2	407.5	412.3	395.4	15.5	20.7	11.4	24.3	16.0
			2	406.3	405.6	408.9	395.3	412.0	18.1	25.1	16.2	21.5	11.7
			3	402.6	411.3	406.1	405.1	407.8	22.6	25.9	16.0	21.8	12.4
			4	407.6	409.4	409.0	382.8	410.4	18.9	23.6	23.6	18.7	15.5
	PS	Vertical	1	1082.4	1050.6	1078.5	1048.1	1073.5	20.3	19.8	25.5	28.1	12.9
			2	1082.7	1067.5	1072.1	1048.2	1085.4	23.2	18.9	16.3	19.8	18.1
			3	1060.7	1069.8	1050.6	1051.7	1067.1	16.8	20.0	19.4	23.8	9.4
			4	1066.3	1084.3	1060.6	1070.9	1067.1	21.2	10.2	20.2	10.7	9.3
		Horizontal	1	1016.8	1012.5	1010.1	987.1	1000.8	21.8	26.9	11.1	22.4	16.0
			2	1003.6	1020.6	999.2	1004.7	1004.8	21.4	26.7	12.0	22.7	9.1
			3	1003.1	994.5	1011.6	1003.8	1016.6	22.5	18.5	13.6	22.4	11.3
			4	1004.2	1019.7	1012.8	1015.6	1001.5	22.8	14.3	20.1	15.7	11.9
Gyroid	ST	Vertical	1	302.3	324.1	304.5	325.3	306.3	14.3	18.0	14.7	21.6	14.1
			2	312.1	306.0	304.3	319.5	318.2	16.9	16.9	17.7	27.1	11.7
			3	316.2	316.1	304.9	318.2	310.3	16.7	22.0	17.2	18.4	11.0
			4	319.3	304.5	319.6	306.5	313.6	20.8	15.2	15.2	15.8	11.3
		Horizontal	1	512.7	491.7	486.3	503.4	489.4	44.4	51.8	33.8	33.4	19.7
			2	497.5	498.9	497.7	499.1	497.6	39.1	39.7	38.5	28.7	23.8
			3	516.0	491.8	506.4	504.4	499.5	34.6	33.5	43.7	22.6	18.3
			4	515.2	480.7	501.0	485.2	498.3	49.1	47.1	47.2	44.1	17.3
	PS	Vertical	1	781.4	771.2	788.8	758.6	784.5	16.5	16.5	14.3	23.5	18.9
			2	781.4	774.9	790.8	769.3	773.2	20.6	17.5	21.5	21.1	10.3
			3	773.3	772.9	781.1	757.9	778.7	24.0	22.7	15.8	21.0	13.0
			4	776.8	793.4	770.0	775.3	771.1	17.4	14.2	16.4	21.0	10.0
		Horizontal	1	606.7	611.0	635.6	643.4	639.3	48.8	37.5	38.1	23.2	15.8
			2	586.1	639.7	656.3	662.1	641.3	53.4	28.7	22.6	20.0	15.1
			3	589.5	611.9	632.2	648.2	636.1	55.3	35.2	29.6	24.3	15.9
			4	595.2	652.8	642.6	653.1	643.1	41.3	39.2	37.0	32.8	16.5

Calculation of s2 in Table 3

The standard deviations s1 in Table 3 describe how much individual measurements in the same slice vary about their slice means, while the standard deviations s2 describe how much individual measurements within a replicate structure vary about the mean for that structure. The values of s2 are naturally larger than s1 because the slice means also vary. They are calculated from the following statistical model. Let y_{ij} denote a measurement of a variable (ST or PS) made at a random location *j* in a chosen slice i of a given orientation in a structure. We can represent this measurement as:

$$y_{ij} = \mu + u_i + e_{ij}$$

where μ is the population mean for that structure, and u_i and e_{ij} are random values drawn from distributions with mean zero and standard deviations σ_u and σ_1, respectively. Thus, u_i describes how the means for different slices vary about the population mean for that replicate structure, and e_{ij} describes how measurements in the same slice a vary about the slice mean. In this model, the variance of y_{ij} is given by:

$$\sigma_2{}^2 = \sigma_u{}^2 + \sigma_1{}^2$$

where σ_2 represents the standard deviation of measurements made at different locations in the structure (whereas σ_1 is the standard deviation of measurements in the same slice). The quantities s1 in Table 3 are estimates of σ_1 for each combination of structure type, variable and orientation, and the quantities s2 are estimates of σ_2 which are calculated as follows. From the model equation, the mean of *n* measurements at random locations in the same slice is given by:

$$\bar{y}_i = \mu + u_i + \bar{e}_i$$

where $\bar{e}_i$ is the mean of the e_{ij} values for that slice, and (according to our model) has variance $\sigma_1{}^2/n$. The variance of $\bar{y}_i$ is therefore:

$$\sigma_u{}^2 + \sigma_1{}^2/n$$

and hence its standard deviation is $\sqrt{(\sigma_u{}^2 + \sigma_1{}^2/n)}$. Furthermore, the standard deviations in Table 3 are estimates of $\sqrt{(\sigma_u{}^2 + \sigma_1{}^2/n)}$, where $n = 30$. From these we can estimate σ_u and hence σ_2. For example, for Diamond ST measurements in vertical slices, the estimate of σ_1 is 18.5 microns, and the estimate of σ_u is given by $\sqrt{(10.52^2 + 18.5^2/30)} = 9.96$ microns. Hence the estimate of σ_2 is $\sqrt{(18.5^2 + 9.96^2)} = 21.0$ microns which is the value of s2 in Table 3. The other values of s2 in Table 3 are calculated in the same way.

Estimating the standard deviations σ_u and σ_1, and hence σ_2, has at least two purposes. First, it allows us to understand better how individual measurements vary and second, it gives us a method of calculating standard errors and confidence intervals of estimates in other situations, particularly when the number of measurements made on each slice, n, differs from 30.
